# Hedgehog pathway is involved in nitidine chloride induced inhibition of epithelial-mesenchymal transition and cancer stem cells-like properties in breast cancer cells

**DOI:** 10.1186/s13578-016-0104-8

**Published:** 2016-06-16

**Authors:** Mingjuan Sun, Ning Zhang, Xiaolong Wang, Yaming Li, Wenwen Qi, Hanwen Zhang, Zengjun Li, Qifeng Yang

**Affiliations:** Shandong Cancer Hospital and Institute, Jiyan Road 440, Jinan, 250117 Shandong Province People’s Republic of China; Department of Breast Surgery, Qilu Hospital, Shandong University, Wenhua Xi Road No. 107, Jinan, 250012 Shandong Province People’s Republic of China; Pathology Tissue Bank, Qilu Hospital, Shandong University, Wenhua Xi Road No.107, Jinan, 250012 Shandong Province People’s Republic of China

## Abstract

**Background:**

The complications of clinical metastatic disease are responsible for the majority of breast cancer related deaths, and fewer therapies substantially prolong survival. Nitidine chloride (NC), a natural polyphenolic compound, has been shown to exhibit potent anticancer effects in many cancer types, including breast cancer. The epithelial-mesenchymal transition (EMT) and the acquisition of cancer stem cells (CSCs)-like properties emerge as critical steps in the metastasis of human cancers. However, the effects of NC on the EMT and the CSCs-like properties in breast cancer cells, and the underlying molecular mechanisms are not fully understood.

**Results:**

In the present study, MDA-MB-468 and MCF-7 cancer cells were treated with NC. Scratch and Transwell assays were performed to determine whether NC could attenuate the migratory and invasive capability of cancer cells; Mammosphere formation and flow cytometry analysis were performed to confirm that NC decreased CSCs-like phenotype; RT-PCR and western blot analysis were used to examine the expression level of EMT and CSC related markers in both cells. Mechanistically, NC could inhibit the components of Hedgehog pathway (smoothened, patched, Gli1 and Gli2), subsequently inhibited the expression of Snail, Slug and Zeb1, which were correlated with the significant changes of the expression of EMT related markers (N-cadherin, E-cadherin, and Vimentin) to reverse EMT. On the other hand, NC could also inhibit the expression of CSCs related factors such as Nanog, Nestin, Oct-4 and CD44 via Hedgehog pathway. Furthermore, transforming growth factor-β1 (TGF-β1)-induced increment of EMT and CSCs properties could be reversed by NC.

**Conclusions:**

Taken together, these data indicated that NC suppressed breast cancer EMT and CSCs-like properties through inhibiting Hedgehog signaling pathway. Our study suggested that NC may be a potential anticancer agent for breast cancer.

## Background

Despite advances have been made in breast cancer treatment over the last decades, this malignancy is still the common cause of cancer-related deaths among females worldwide. Presently, breast cancer has been one of the most commonly diagnosed cancers, with approximately 1.7 million new patients in 2012, accounting for 25 % of all women cancer cases [1]. And the majority of cancer deaths can be due to development of related metastatic disease. In spite of the initial effectiveness of conventional therapies, recurrence and the emergence of metastases are major causes of therapeutic failure in cancer patients. The relatively high relapse and metastasis rate of patients with aggressive forms of breast cancer are attributed to a small population of cancer stem cells (CSCs) or tumor-initiating cells (TICs) residing within tumor. Recently, CSCs, together with epithelial mesenchymal transition (EMT)-type cells which shares similar molecular characteristics with CSCs, have been demonstrated to play vital roles in tumor metastasis. They also contribute to radioresistance and chemoresistance as reported in several malignancies such as breast cancer. Therefore, novel adjuvant agents with complete efficacy and low toxicity are directed to be applied in the treatment of metastatic breast cancers and improve patient survival.

Nitidine chloride (NC), which is a natural phytochemical alkaloid, is isolated from the root of Zanthoxylum nitidum, and possesses pleiotropic anticancer capabilities in different cancer types, including breast cancer. It has been found that NC exhibits several types of pharmacologic properties closely related to health therapies, including antimalarial [2], anti-inflammatory [3], antifungal [4], antiangiogenesis [5], and anticancer activity [6]. It has been reported that NC can inhibit the ability of cellular migration and invasion in breast cancer through suppressing the c-Src/focal adhesion kinase (FAK)-associated signaling pathway. Our previous study has demonstrated that NC can cause cell cycle arrest and apoptosis by regulating cyclin-CDK cascade, caspase 3 and PARP cleavages in breast cancer cells [7]. Interestingly, the combination of NC and doxorubicin has a synergistic inhibitory effect on cellular proliferation of breast cancer [7]. However, the underlying mechanism of NC in regulating cancer cellular EMT and CSCs-like phenotype are little investigated.

Metastasis occurring involves a complex and multistep process, including cellular invasion from primary tumors into the circulation, their migration to distant organs, then adhesion to endothelial cells and infiltration into tissues. Accumulating evidences suggest that EMT can play a critical role in tumor invasion and metastasis by equipping these cells with a more motile and invasive phenotype [8–10]. Several signaling pathways like the TGF-β, Wnt, Notch and Hedgehog pathways [11, 12], have been involved in mediating these transitions, through regulating the expression of crucial EMT-related transcription factors, such as Snail, ZEB and Twist families [13, 14]. In recent years, there has been growing studies that induction of EMT phenotype by different factors results in the enrichment of cells with stem-like characters, termed cancer stem cells [15–17]. The population of CD44^+^/CD24^−/low^ has emerged as one of the key markers for isolated breast cancer stem cell, which have been reported to be associated with secondary metastasis and resistance to radiotherapy on breast cancer tissues [18, 19]. The complex relationship between EMT and stemness phenotype has acquired huge interest in the field of cancer research.

Here, we showed that NC could suppress breast cancer metastasis. And the anti-cancer effects of NC were due to decrease of the Hedgehog signaling. Subsequently, NC showed the inhibitory effect on the expression of transcription factors (Snail, Slug and Zeb1), and then reversed EMT with decreased mesenchymal markers (i.e., N-cadherin and Vimentin) and increased epithelial markers (E-cadherin), meanwhile it inhibited the expression of pluripotency maintaining factors including Nanog, Nestin and Oct-4, and then suppressed CD44 expression and CSC phenotypes, all of which were crucial for distant metastasis of breast cancer.

## Methods

### Cell culture and NC treatment

Breast cancer cell lines MCF-7 and MDA-MB-468 were obtained from American Type Culture Collection (ATCC, Manassas, VA, USA), and were routinely cultured in DMEM medium (GIBCO Laboratories, Grand Island, NY, USA) supplemented with 10 % FBS (Haoyang biological manufacture, Tianjin, China), 100 U/ml penicillin and 100 mg/ml streptomycin. Nitidine chloride was obtained from Tauto Biotech (Shanghai, China) and dissolved in dimethyl sulfoxide (DMSO). Epidermal growth factor (EGF), basic fibroblast growth factor (bFGF), and bovine insulin were obtained from Sigma Aldrich (St. Louis, MO, USA). B27 supplement (50×) was purchased from Gibco. For NC treatment, stock solution (5 mM in DMSO) was added into culture medium to achieve appropriate concentration (1 or 2.5 μM), and then incubated with cells, whereas DMSO solution without NC was used as a control.

### Reagents and antibodies

The Hedgehog inhibitor, cyclopamine was purchased from Selleck Chemicals (Houston, USA). TGF-β1was purchased from sigma (St.Louis, MO, USA). Primary antibodies against E-cadhein, N-cadherin, Snail, and Vimentin were procured from cell signaling technology (Beverly, MA, USA). The anti-CD44, anti-Gli-1, anti-Gli-2, anti-Patched and anti-Smoothened antibodies were obtained from Immuno-way (Newark, DE, USA). Anti-β-actin was purchased from Dako Corp (Carpinteria, CA, USA). The secondary horseradish peroxidase (HRP)-linked antibodies were obtained from Tiangen Biotech CO., LTD (Beijing, China).

### Cell proliferation assay by MTT assay

The effect of NC on proliferation of MDA-MB-468, MCF-7 parental cells and mammospheres was examined by MTT [3-(4, 5-dimethylthiazol-2-yl)-2, 5-diphenyltetrazolium bromide] assay. Cells (2.5 × 10^3^ per well) were seeded in 96-well plates and allowed to grow for 24 h. Then NC or DMSO (vehicle control) was added after plating onto adherent cells at specified concentrations. After indicated time of treatment, 20 μl of MTT solution (5 mg/ml; Sigma, St. Louis, MO) was added to each well and incubated for 4 h at 37 °C. The MTT formazan crystal was then dissolved in DMSO, and the absorbance was measured by a Microplate reader (Bio-Rad 680, Bio-Rad Laboratories, Hercules, CA) at a wavelength of 570 nm.

### In vitro scratch assay

Cell migratory ability was detected by a wound-healing assay. The MDA-MB-468 and MCF-7 cells were seeded in 24-well plates (1.0 × 10^5^ cells/500 μl). After the cells grew to 90–100 % confluence, a 200 μl sterile pipette tip was used to produce a wound line between the cells. Cellular debris was removed by washing with PBS. The wounded monolayers were then incubated with NC or vehicle for 24 h. The distance between two cell edges were analyzed by ImageJ software.

### Migration and invasion assay

The migration and invasion of breast cancer cells were performed in transwell chambers (24-well, 8 μm pore size with polycarbonate membrane; Corning Costar, Lowell, MA, USA). The invasion assay was performed in the same way as the migration assay except that the membrane was coated with matrigel (BD Biosciences, Bedford, MA, USA). Briefly, the polycarbonate membranes were coated with 40 ml matrigel at 37 °C for 2 h to form a reconstituted basement membrane. Seven hundred microlitres of the medium with 20 % FBS was added to the lower well of each chamber and 1 × 10^5^ of cells resuspended in the serum-free medium were added to the upper inserts. The total number of cells adhering to the lower surface of the membrane was acquired in six representative fields.

### Spheroid formation

Tumorsphere formation assay was carried out as previously described [20]. Briefly, suspension single cells were plated at a density of 10,000 cells/ml and grown in a serum-free DMEM/F12 medium (Gibco-BRL, Rockville, IN, USA), supplemented with 5 μg/ml bovine insulin, 20 ng/ml EGF, 20 ng/ml bFGF, and 1 × B27 supplement. Cells were grown for 7–14 days, and medium was replenished every 3–4 days. Tumorsphere volume was calculated using the following formula: (length × width^2^)/2. The numbers of spheres with diameter over 50 μm were counted through a microscope.

### Flow cytometry analysis

Cell surface markers were detected by flow cytometry analysis following previous protocols [21]. Briefly, cells were harvested and resuspended with Pharmingen Stain Buffer to a final concentration of 2 × 10^7^ cells/ml. Then, fluorochrome conjugated mouse anti-human CD44 or CD24 in combination with their respective isotype controls was added and incubated for 20 min on ice, protected from light. After being washed twice, the cells were resuspended in 0.5 ml Stain Buffer and analyzed by a FACScan flow cytometer (Becton–Dickinson, Franklin lakes, NJ, USA).

### Quantitative real-time PCR

Total RNA was extracted from cultured cells with TRIzol reagents (Invitrogen) according to the manufacturer’s protocol. Briefly, Total RNA was reverse transcribed to cDNA by using Prime Script RT reagent Kit (Takara, Dalian, China). Real-time PCR was performed with the SYBR green Premix Ex Taq II (Takara) with Applied Bio-systems Step One Plus Real-Time PCR System (Applied Bio-systems, Carlsbad, CA, USA). Relative quantification analysis was performed using the comparative C (T) (2(−ΔΔCT)) method. The following gene-specific primers were used. *GAPDH* (5′-AGA AGG CTG GGG CTC ATT TG-3′, 5′-AGG GGC CAT CCA CAG TCT TC-3′); *Snail* (5′-ACC CCA CAT CCT TCT CAC TG-3′, 5′-TAC AAA AAC CCA CGC AGA CA-3′); *Slug* (5′-ACA CAC ACA CAC CCA CAG AG-3′, 5′-AAA TGA TTT GGC AGC AAT GT-3′); *Zeb1* (5′-GCA CAA CCA AGT GCA GAA GA-3′, 5′-CAT TTG CAG ATT GAG GCT GA-3′); *E-cadherin* (5′-GAA GCA CAG AAT CCC CAA GTG-3′, 5′- GGC GTG TTT GTC TTC CAT TTC-3′); *Vimentin* (5′-GAC AAT GCG TCT CTG GCA CGT CT-3′, 5′-TCC GCC TCC TGC AGG TTC TT-3′); *Twist* (5′-CAA GTC TGC AGC TCT CGC CA-3′, 5′-CCA ACG GCT GGC GCA CAC-3′); *Nestin* (5′-AAC AGC GAC GGA GGT CTC TA-3′, 5′-TTC TCT TGT CCC GCA GAC TT-3′); *Nanog* (5′-ACC TAC CTA CCC CAG CCT TT-3′, 5′-CAT GCA GGA CTG CAG AGA TT-3′); *Oct4* (5′-GGA CCA GTG TCC TTT CCT CT-3′, 5′-CCA GGT TTT CTT TCC CTA GC-3′); *Smoothened* (5′-TCG CTA CCC TGC TGT TAT TC-3′, 5′-GAC GCA GGA CAG AGT CTC AT-3′); *Gli1* (5′-CTG GAT CGG ATA GGT GGT CT -3′, 5′-CAG AGG TTG GGA GGT AAG GA-3′).

### Western blot analysis

Cell indicated were treated with NC for 48 h, and then total cellular protein lysates were prepared with radio immunoprecipitation assay buffer (PBS, 1 % NP40, 0.1 % SDS, 5 mM ethylenediaminetetraacetic acid, 0.5 % sodium deoxycholate, 1 mM sodium orthovanadate) containing proteinase inhibitors. A total of 30–40 μg of protein was separated by SDS-PAGE and transferred to a polyvinylidene difluoride membrane (Millipore, Bedford, MA, USA). Following blocking with 5 % non-fat milk, the membranes were incubated overnight at 4 °C with the primary antibodies. After being washed with TBST, membranes were incubated with secondary antibodies at room temperature for 2 h, and the proteins were detected by a chemiluminescence system (ECL kit). Loading differences were normalized using a monoclonal β-actin antibody.

### Statistical analysis

All assays were repeated in triplicates in three independent experiments, and quantitative data were presented as mean ± SD. The differences between two groups were compared by Student’s t test. All error bars represent the S.E. of three experiments. In all cases, P < 0.05 was considered statistically significant. All statistical analyses were performed using SPSS 18.0 (SPSS Inc., Chicago, IL, USA).

## Results

### NC inhibited the proliferation of breast cancer parental cells and mammospheres

**Fig. 1:**
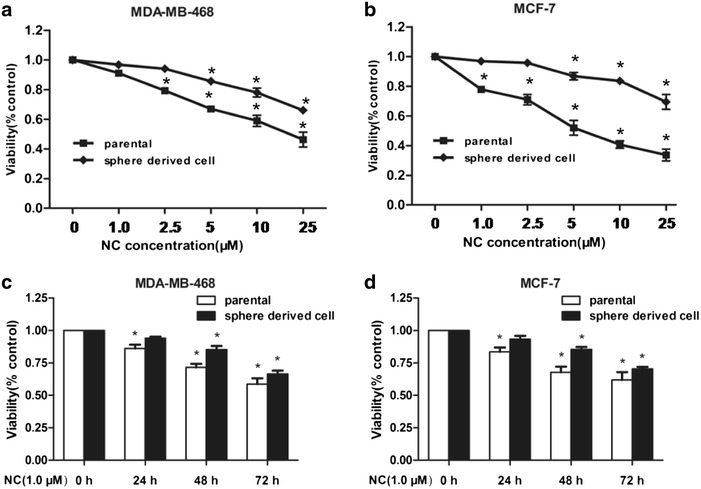
The effect of NC on breast cancer parental cells and mammospheres viability was measured using MTT based. **a**, **b** NC had effects on cell proliferation of breast cancer MDA-MB-468 and MCF-7 parental cells and mammospheres in a concentration dependent manner. However, sphere-derived cells showed relative resistance to NC compared to parental cells. **c**, **d** Both Parental cells and mammospheres were treated with indicated concentrations of NC. However, no obviously cell death was observed by NC at 1.0 μM for 24, 48 and 72 h in MTT assay. *P < 0.05 compared with the control. The data are presented as the mean ± SD of three separate experiments

The proliferation-inhibitory effects of NC with different concentrations on MDA-MB-468 and MCF-7 cell lines were investigated by MTT assay. Both results indicated NC-induced inhibition of MDA-MB-468 and MCF-7 parental cells proliferation in a dose-dependent manner at 48 h (Fig. 1a, b). To determine whether NC could inhibit the viability of cancer stem cells, MDA-MB-468 and MCF-7 mammospheres were used as a model of breast CSCs. As shown in Fig. 1c, d, the viable mammospheres at 24, 48, and 72 h after the treatment with NC (1.0 μM) were decreased time-dependently. However, mammospheres showed more resistance to the NC-induced inhibition effect compared to parental cells.

### NC inhibited the migration and invasion of breast cancer cells

**Fig. 2:**
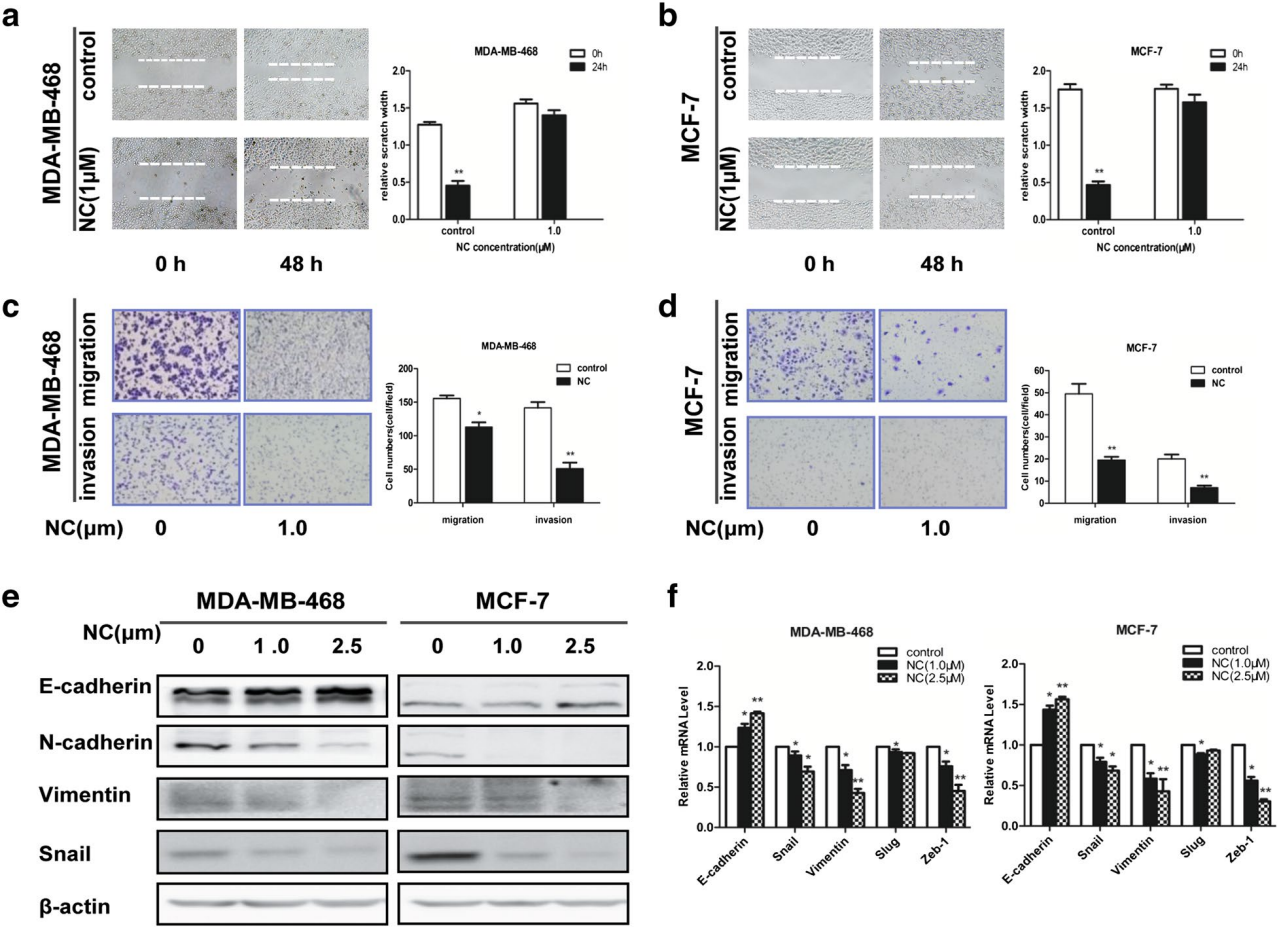
NC attenuates the migratory capacity and EMT of breast cancer cells. **a**, **b** MDA-MB-468 and MCF-7 cells were treated with the indicated concentrations of NC for 48 h. NC significantly inhibited the migration of cells into the denuded areas. The MDA-MB-468 and MCF-7 cells were treated with increasing concentrations of NC (0, 1.0 μM) for 48 h. **c**, **d** Photos of the invasive and migration were taken under a microscope with a 200× objective. **e**, **f** Both cell types were treated with 0, 1, or 2.5 μM NC for 48 h and then Protein and mRNA expression levels of EMT-related genes were detected. *P < 0.05 or **P < 0.05 compared with the control group

The effect of NC on cellular motility of breast cancer was determined by using wound-healing assay. Serum starved MDA-MB-468 and MCF-7 cells in the control group exhibited marked cell migration in the wound area after 48 h wounding, while the cells treated with NC (1.0 μM) showed relative delays in wound closure (Fig. 2a, b). To examine its potential anti-invasive effects, the ability of invasion was analyzed in MDA-MB-468 and MCF-7 cells treated with NC. As shown in Fig. 2c and d, the average number of cells invading into the lower chamber decreased accordingly, when the NC concentration increased from 0 to 1.0 μM. Since 1.0 μM of NC decreased the cellular migratory and invasive capability of breast cancer effectively without obvious cytotoxicity (Fig. 1a, b), we chose this concentration for further investigation. This finding revealed that NC might be an effective inhibitor of cellular migration and invasion of breast cancer.

### NC inhibited EMT (epithelial-mesenchymal transition) in breast cancer cells

It is believed that acquiring the migratory characteristics of a mesenchymal-like state can enhance the invasive capabilities of cancer cells. EMT is crucial for the invasion and metastasis of breast cancer, and we have firstly showed that NC could inhibit cell migration and invasion of breast cancer MDA-MB-468 and MCF-7 cells, which was accompanied with upregulation of epithelial marker E-cadherin and downregulation of mesenchymal markers N-cadherin and Vimentin at the mRNA level (Fig. 2f). As expected, NC could also inhibit the expression of the transcription factors Snail, Slug and Zeb-1 at the mRNA level (Fig. 2f).

The protein expression of E-cadherin, N-cadherin, Vimentin and Snail in the breast cancer cells subject to NC at 48 h were evaluated by Western blotting. As shown in Fig. 2e, NC treatment could also influence the expression of EMT-related factors at protein levels, and this trend was consistent with the mRNA results.

### NC decreased the CSCs-like properties of breast cancer cells

**Fig. 3:**
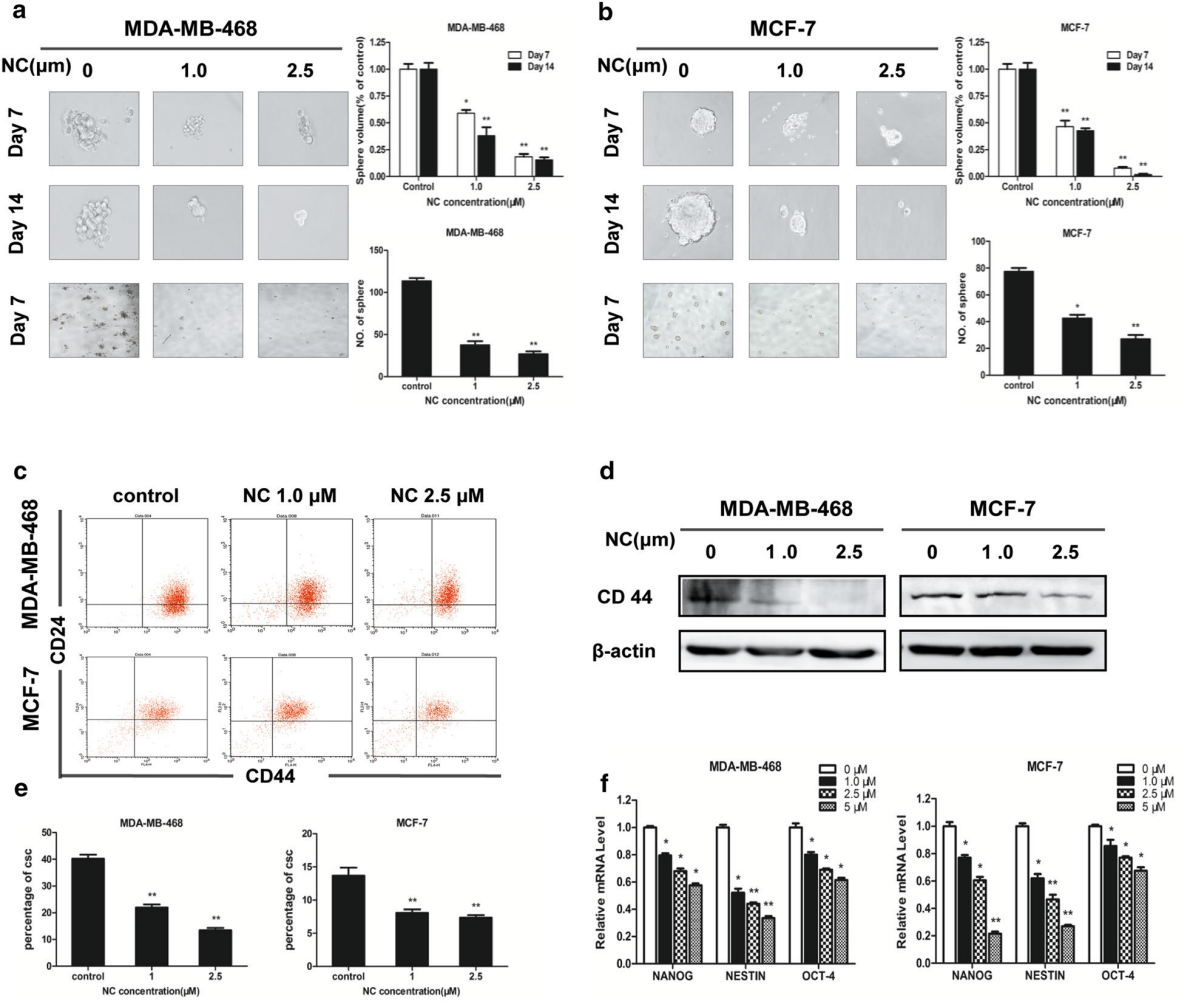
NC decreases the CSCs-like properties of breast cancer cells. NC inhibited mammosphere formation of MDA-MB-468 (**a**) and MCF-7 (**b**) cell lines. NC reduced the number of mammospheres after 7 days of treatment. Smaller sizes of mammospheres were observed in comparison with control. Representative images of* spheres* were shown above. The MDA-MB-468 and MCF-7 cells were treated with increasing concentrations of NC (0, 1.0 to 2.5 μM) for 48 h. **c** flow cytometry was used to detect cell surface markers. A set of representative flow cytometry *plots* was shown above. **e** NC significantly suppressed the number of both cells expressing CD44^+^/CD24^−/low^ in a dose-dependent manner. **d** The expressions of CD44 were observed by western blotting. **f** The expressions of Oct-4, Nestin, and Nanog were determined by real-time PCR with GAPDH as a loading control. Experiments were independently repeated at least three times

Development of EMT involves acquisition of stem cell-like features, including the surface marker expression of stem cells, nonadherent growth, as well as changes in expression of cell-surface glycoproteins [15]. The subpopulation of CD44^+^/CD24^−/low^ cells has been shown to exhibit cancer stem-like properties, and these cell surface markers have been used as functional markers for sorting cancer stem cells from parental cell lines [22]. In our present study, NC significantly suppressed the number of MDA-MB-468 and MCF-7 cells expressing CD44^+^/CD24^−/low^ in a dose-dependent manner (Fig. 3c, e). Consistently, after treatment with NC for 48 h, a decreased expression of CD44 protein was observed (Fig. 3d). Moreover, formation of spheroids demonstrates cellular capacity for self-renewal and for initiation of tumors, both of which are characteristics of cancer stem cells (CSCs) [23]. We then investigated the effects of NC on the formation of spheroids in breast cancer cells. In nonadherent dishes, breast cancer cells formed free-floating and viable of spheres; however, after 48 h NC treatment, such characteristics of these cells were abolished. As shown in Fig. 3a, b, not only was the volume of mammospheres significantly reduced, but the number of mammospheres was significantly declined in time-and does- manner. These results were consistent with those from the MTT assay.

As is known, Nanog, Nestin and Oct-4 are required for maintaining pluripotency in stem cells [24, 25]. Therefore, we further examined the effects of NC on these factors. As shown in Fig. 3f, the expression levels of Nanog, Nestin and Oct-4 were inhibited by NC in a dose-dependent way. These data demonstrated that NC could decrease the CSCs like properties of breast cancer cells.

### NC inhibited Hedgehog signaling pathway

**Fig. 4:**
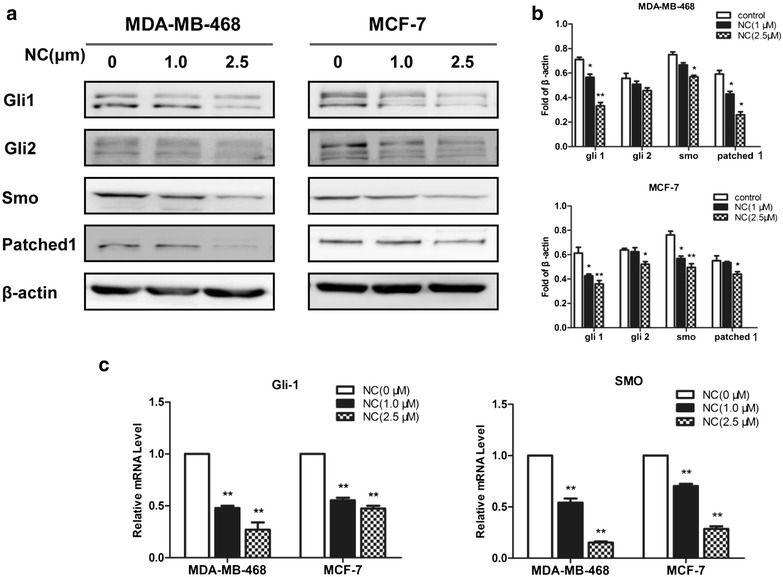
NC inhibited Hedgehog pathway in MDA-MB-468 and MCF-7 cell lines. Cells were treated with various concentrations of NC for indicated time. **a** The Gli1, Gli2, Smo and Patched1 proteins were examined by western blot in both cells. **b** Treatment of both cell lines with NC (0, 1.0, or 2.5 μM) for 48 h resulted in a significant inhibit the activation of Hedgehog signaling pathway in a dose-dependent manner, β-actin was used as a loading control. **c** Then, the expressions of Gli1 and Smo were determined by real-time PCR with GAPDH as a loading control. Results are presented as mean ± SD

The Hedgehog (Hh) signaling pathway is definitely essential for the development of tissues and organs [26]. However, aberrant activation of Hedgehog signaling pathway plays important roles in tumorigenesis and progression of several tumors [27]. We hypothesized that Hedgehog signaling pathway might as well be implicated in the NC-induced inhibition of EMT and CSCs-like properties in both breast cancer cells. We first sought to examine the effects of NC on Hedgehog pathway by measuring the expression of Hh receptors (Patch, Smo) and effectors (Gli1, Gli2) by western blot (Fig. 4a). As shown in Fig. 4b, NC downregulated the expression of Smo and Patch. Similarly, NC inhibited the expression of transcription factor Gli1 and Gli2, and its inhibition effecton Gli1 expression was much more significant than Gli2. Then, we examined the effects of NC on mRNA expression of Gli1 and Smo by RT-PCR in breast cancer cell line MDA-MB-468 and MCF-7. As shown in Fig. 4c, NC inhibited the mRNA expression of Gli1 and Smo in these cells as well, suggesting that NC could inhibit the activation of Hh signaling in breast cancer.

### Hedgehog were involved in the NC-induced inhibition of EMT and CSCs-like properties

**Fig. 5:**
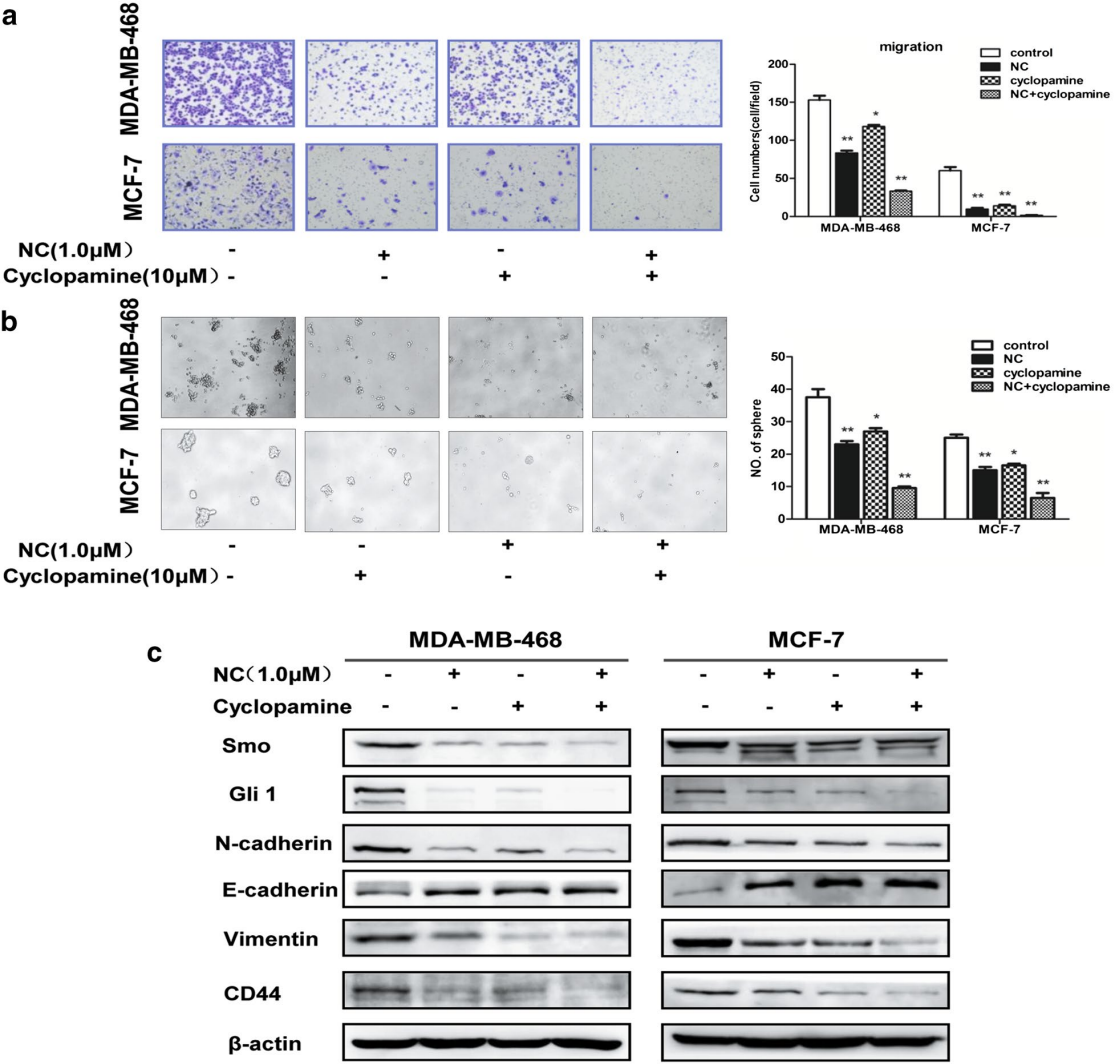
NC induced inhibition of in breast cells by Hedgehog-Gli1 pathway. After both cells were pretreated by cyclopamine (a Smo inhibitor) for 6 h, they were exposed to 0, 1, or 2.5 μM of NC for 48 h respectively. **a** Transwell assay analyses of the migratory capacity of both cells. Then quantification of transwell cell migration assay. **b** Free-floating, viable* spheres* formed by breast cancer cells. **c** Western blotting analyses of Gli1, Smo, E-cadherin, N-cadherin, Vimentin, CD44 levels. Blots were normalized by use of β-actin to correct for differences in loading of the proteins.*P < 0.05 or **P < 0.01. The data are presented the mean ± SD of three separate experiments

To further confirm whether NC is Hedgehog dependent, we inhibited Hedgehog pathway using the Smo inhibitor Cyclopamine [28, 29]. After pretreatment of MDA-MB-468 and MCF-7 cells with 10 μM of Cyclopamine for 6 h, both cells were subject to 0 or 1.0 μM of NC for 48 h respectively. We found that in both presence of NC and Cyclopamine the migratory capacity of breast cancer cells was further decreased, compared to the treatment of NC only (Fig. 5a). As shown in Fig. 5a, c, blockade of Hh signaling pathway by Cyclopamine could significantly enhance NC-induced inhibition of Hh and Gli1 expression as well as inhibition of migration of breast cancer cells. It has been reported that Snail induction and E-cadherin suppression were early responses to Gli1 overexpression [30], which is similar to our following results that Cyclopamine could down-regulate N-cadherin and Vimentin expression and up-regulate E-cadherin expression induced by NC (Fig. 5c).

Emerging data from many human tumors have manifested that Hedgehog–Gli1 signaling could regulate CSCs activities [31]. Furthermore, we observed the effects of Hedgehog on NC-induced inhibition of CSCs-like properties in breast cells, and cells were treated as described above. NC could inhibit the formation of spheroids in our present study. However, inhibition of Hh pathway could enhance the effect above induced by NC (Fig. 5b). As shown in Fig. 5c, Cyclopamine inhibited the expression of Smo and further attenuated the expression of transcription factor Gli1. Interestingly, in the presence of Cyclopamine and NC, inhibition of Hh-Gli1could further decrease the expression of CSCs related marker CD44 compared to the NC alone. These results indicated that Hedgehog was involved in NC-induced inhibition of EMT and CSCs-like properties in breast cancer cells.

### NC suppressed TGF-β1 induced EMT and CSC of breast cancer cells

**Fig. 6:**
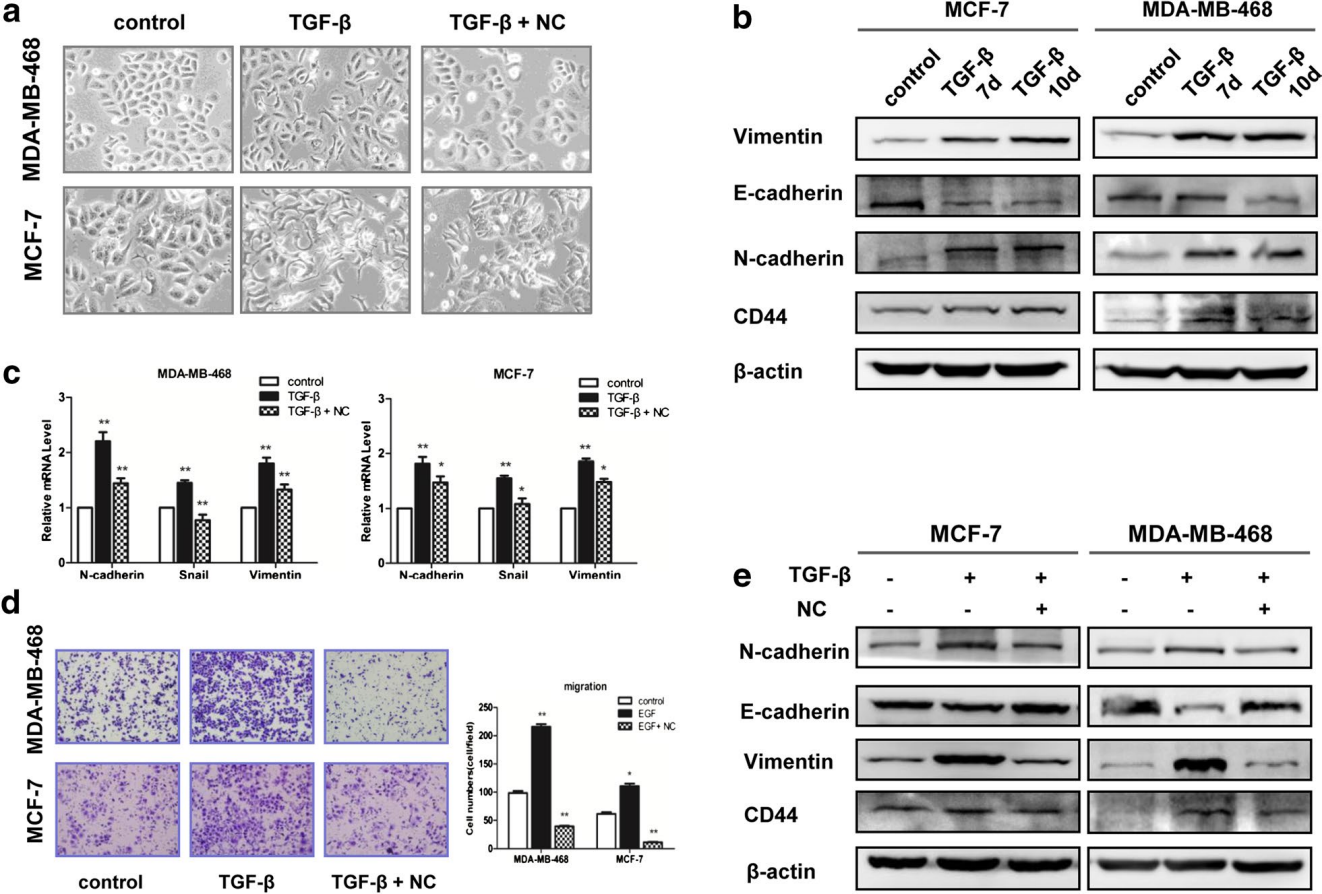
The effects of NC on the TGF-β-induced EMT and CSCs-like properties of breast cancer cells. MDA-MB-468 and MCF-7cells were treated with 5 ng/ml TGF-β1 with or without 2.5 μM NC for 48 h. **a** Cells were treated with, and the phenomenon of EMT was observed based on morphological changes of the cells. **b** The both cells were treated with5 ng/ml TGF-β for 7 d or 10 d. The expressions of EMT and CSCs-related marks were observed by western blotting. β-actin was used as a loading control. **c** Treatment with NC for 48 h diminished the effects of TGF-β1 on the expression of N-cadherin, Vimentin at the mRNA level in MDA-MB-468 and MCF-7cells, as determined by QT-PCR. **d** The effects of NC in breast cancer cell migration. The number of migrated cells was quantified by counting the number of cells from 5 random fields. **e** Treatment with NC for 48 h diminished the effects of TGF-β1 on the expression of EMT and CSCs-related marks at the protein level in both cells, as determined by western blotting

Previous studies have revealed that TGF-β plays a critical role in driving EMT process in the pathogenesis of breast cancer [32, 33]. We next examined whether NC could inhibit TGF-β1 induced EMT, which is associated with cellular migratory and invasive ability. Morphological changes of MDA-MB-468 and MCF-7 cells after TGF-β1 treatment were followed in the absence or presence of NC, which was observed by optical and confocal microscopy. As shown in Fig. 6a, addition of TGF-β1 at concentration of 5 ng/ml for 72 h altered the morphology of both cells from an epithelial phenotype to a classical mesenchymal phenotype. Cells treated with 5 ng/ml TGF-β1 and 2.5 μM NC displayed classical epithelial morphology. The breast cancer cell line has been demonstrated to undergo EMT process in response to TGF-β1 stimulation [34]. To ensure whether this is also the case for MDA-MB-468 and MCF-7, we assessed the expression of various EMT-associated markers and the CSC-related markers after 7 d and 10 d treatment with TGF-β1 (Fig. 6b).

To further confirm the effect of NC on TGF-β1 induced EMT and CSCs properties, we determined the expression levels of EMT related marks after the cells were treated with 5 ng/ml TGF-β1 with or without 2.5 μM of NC. As shown in Fig. 6c, TGF-β1 up-regulated the mRNA level of N-cadherin and Vimentin as mesenchymal marker. NC significantly reversed all of these TGF-β1 induced effects. EMT is thought to be a prerequisite for tumor cells to become motile and eventually more invasive and metastatic. In order to determine the potential role of NC in cellular metastasis of breast cancer by inhibiting TGF-β1-induced EMT, transwell assays were conducted to observe the effects of NC on cell migration. The results showed that TGF-β1 markedly enhanced the migratory ability of the cells (Fig. 6d), and NC reversed these effects. To evaluate the effects of NC on the expression of E-cadherin, N-cadherin, Vimentin, Snail and CD44 at protein level, we determined these proteins in MDA-MB-468 and MCF-7 cells with or without NC using Western blotting. As shown in Fig. 6e, NC could down-regulate protein expressions of TGF-β1 induced EMT and CSC related markers, and the trend was consistent with the mRNA results. Taken together, our results demonstrated that NC could suppress TGF-β1 induced EMT and CSC in both breast cancer cells.

## Discussion

NC has recently received more attention for its pleiotropic anticancer roles in inhibition of tumor angiogenesis, inflammatory, and inhibition of proliferation and metastasis [3, 5, 6, 35]. Previous evidence has suggested that NC has the potential to prevent breast cancer development, for example, NC induces breast cancer cell apoptosis though the intrinsic, mitochondrial-dependent caspase pathway [7]. In addition, combined with doxorubicin, NC could exhibit a synergistic inhibitory effect on cellular growth of the human breast cancer cell lines. In the present study, we first reported that NC exhibited a chemotherapeutic effect against EMT and CSCs, and then abrogated breast cancer metastasis. After exposure of breast cancer cells to NC, they showed inhibited metastasis ability of breast cells by inhibiting the expression of transcription factors (Snail, Slug and Zeb1) and EMT markers. What is more, NC could inhibit cellular CSCs-like self-renewal capacity of breast cancer by inhibiting the expression of pluripotency maintaining transcription factors (Nanog, Nestin and Oct-4) and CSCs markers (CD44) as well as the components of Hh pathway. In addition, our results demonstrated that NC also suppressed TGF-β1 induced EMT, expression of CSCs-related markers as well as cellular migration in both cell lines. Furthermore, we revealed that these effects were ascribed to the inhibition of Hedgehog pathway by NC treatment on MDA-MB-468 and MCF-7 cells.

EMT is an available strategy to target cancer metastasis, and the loss of E-cadherin and gain of Vimentin have been known to play vital roles in EMT process of various malignancies, breast cancer included. The transcriptional repressors including Snail, Slug, ZEB-1 and ZEB-2 are known to downregulate E-cadherin expression [36, 37]. Furthermore, transformed epithelial cells can activate embryonic programs of epithelial plasticity through switching from a sessile, epithelial phenotype to a motile, mesenchymal phenotype [38]. In the present study, after exposure of breast cancer cells to NC, they showed an inhibited migration and invasion ability. Further, there were increased expression of epithelial marker E-cadherin, and decreased expression of mesenchymal marker N-cadherin and vimentin, as well as down-regulated expression of Snail, Slug and Zeb1 (transcription factors). These results demonstrated that, with exposure to NC, both breast cancer cells undergo a MET.

CSC is believed to be the origin of metastatic breast cancer, which has the capabilities for self-renewal and differentiation into the bulk cancer cells [39]. Acquisition of CSCs-like properties emerges as a key step in cancer progression [40, 41]. TICs and CSCs share a variety of properties, including self renewal, although it is typically unregulated in tumor generation. Many cultured CSC lines form free floating spherical clusters of viable cells with a preponderance of the CSCs [23, 42]. In our previous study, breast cancer cells with NC treatment exhibited suppressed capacity for forming tumorsphere. Many genes were expressed in breast CSCs, including CD44, ALDH etc. [43]. Nanog, Oct-4 and Nestin can co-occupy and regulate their own promoters as well as other developmental genes with diverse functions, and they collaborate to form an extensive regulatory circuitry including auto regulatory and feed-forward loops [44]. This study indicated NC could decrease CD44^+^/CD24^−/low^ subpopulation and suppress the expression of Nanog, Oct-4 and Nestin in both cell lines. In addition, expressions of CD44 were substantially decreased in NC-treated cells of MDA-MB-468 and MCF-7. These results indicated that breast cancer cells could lose CSCs-like characteristics after exposure to NC.

EMT can enrich cells with stem-like properties, while CSCs exhibit mesenchymal phenotypes [15, 45]. Many signaling pathways, including Hedgehog, Notch and Wnt, are believed involving in the maintenance of EMT and CSCs [8]. The Hh signaling pathway has been reported to be associated with cellular migration, invasion and self-renewal ability in several types of cancers [46, 47]. Hh signaling is activated by binding the secreted Hh peptide to the 12-span transmembrane spanning receptors (Ptch), resulting in loss of Ptch activity and consequent phosphorylation and post transcriptional stabilization of smoothened (Smo) [48, 49]. As a result, expression of Hh target genes is initialized through posttranslational activation of the Gli family of zinc-finger transcription factors (Gli1, Gli2, and Gli3) [30]. Our previous study has demonstrated that Hedgehog pathway could play a crucial role in the maintenance of breast CSC properties [21]. In this study, we observed the inhibitory effects of canonical Hedgehog signaling pathway after NC treatment, which was supported by decreased protein expression of transcription activator Gli1 and receptor Patch, and target genes of the Hh signaling pathway. To confirm the effect of NC on Hh, we blocked Hedgehog pathway in both cell lines using the Hedgehog inhibitor Cyclopamine, which inhibited Hh signaling pathway by binding to Smo. Subsequently, Cyclopamine blocked the functions of Gli1 activators, consistent with the expected down-regulation of the Hh–Gli1 Pathway. Further, we found that Cyclopamine could enhance NC-induced reversion of EMT and attenuation of CSC characteristics, as indicated by migration, mammospheres and western blot analysis. Here we found that the Hedgehog pathway was also involved in NC-induced inhibition of EMT capacity and CSCs-like properties in breast cancer cells.

In human breast cancer, the CD44^+^/CD24^−/low^ CSCs signature was linked with a mesenchymal, migratory phenotype. Even more, TGF-β induced EMT may render breast cancer cells with properties of stem cells and even therapy resistance [15, 50]. The TGF-β growth factor plays a complex and important role in EMT induction via canonical and noncanonical TGF-β signaling systems [40]. During the process of cancer metastasis, tumor cells, that metastasize, acquire capabilities of self-renewal, similar to which exhibit in stem cells in order to spread the metastases [37]. In agreement with previous reports [51, 52], we showed that TGF-β1 could induce EMT in MDA-MB-468 and MCF-7 cells, which included acquisition of mesenchymal phenotype, decreased expression of epithelial markers E-cadherin and increased expression of mesenchymal marker Vimentin and N-cadherin, as well as increased expression of CSC markers such as CD44. Similar results could be observed while detecting the E-cadherin and Vimentin mRNA levels by RT-PCR. Moreover, we found that NC could inhibit exogenous TGF-β1-induced EMT and migration, accompanied with the decreasing expression of EMT and CSC-related markers in breast cancer cells.

## Conclusions

**Fig. 7:**
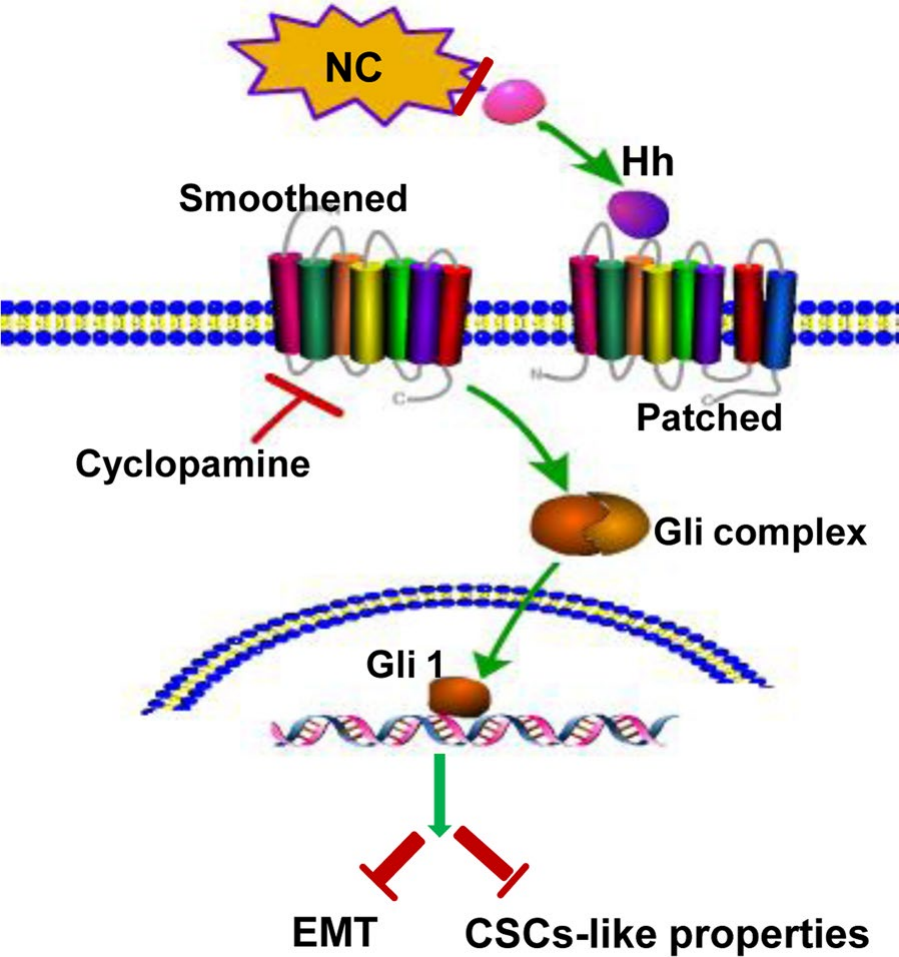
Hedgehog pathway is involved in NC-induced inhibition of EMT and CSCs-like properties in breast cancer cells. In MDA-MB-468 and MCF-7 cells exposed to NC, the schematic *picture* shows the effects of NC dual-blocking EMT and CSCs-like properties and then targeting breast cancer migration and invasion through inactivation of Hedgehog signaling

As summarized in Fig. 7, our studies revealed a novel mechanism involving NC targeting breast cancer metastasis, in which inactivation of Hedgehog signaling pathway by NC treatment led to a significant decreased expression of Smo and Gli1. Suppression of Hh-Gli1 pathway could not only directly reverse EMT, but also inhibit CD44 expression and abolish CSC properties. Furthermore, we reported that the induction of EMT by TGF-β1 treatment in parent breast cancer cells resulted in the acquisition of mesenchymal morphology and up-regulation of the expression of CSCs markers. Moreover, our study showed that NC could also inhibit TGF-β1-induced breast cancer cell metastasis. Therefore, our findings demonstrated that NC could be a novel potential agent for prevention and treatment of breast cancer through dual-blocking EMT and CSCs-like properties.
